# Summary of drug therapy to treat cognitive impairment-induced obstructive sleep apnea

**DOI:** 10.3389/fncel.2023.1222626

**Published:** 2023-09-04

**Authors:** Daqiang He, Jian Chen, Xiaoxue Du, Linhao Xu

**Affiliations:** ^1^Department of Laboratory Medicine, Affiliated Hangzhou First People’s Hospital, Zhejiang University School of Medicine, Hangzhou, Zhejiang, China; ^2^School of Basic Medical Sciences and Forensic Medicine, Hangzhou Medical College, Hangzhou, Zhejiang, China; ^3^Key Laboratory of Clinical Cancer Pharmacology and Toxicology Research of Zhejiang Province, Translational Medicine Research Center, Affiliated Hangzhou First People’s Hospital, Zhejiang University School of Medicine, Hangzhou, China; ^4^Department of Cardiology, Affiliated Hangzhou First People’s Hospital, Zhejiang University School of Medicine, Hangzhou, Zhejiang, China

**Keywords:** obstructive sleep apnea, intermittent hypoxia, sleep fragmentation, cognitive impairment, drug therapy

## Abstract

Obstructive sleep apnea (OSA) is a severe sleep disorder associated with intermittent hypoxia and sleep fragmentation. Cognitive impairment is a signifi- cant and common OSA complication often described in such patients. The most commonly utilized methods in clinical OSA treatment are oral appliances and continuous positive airway pressure (CPAP). However, the current therapeutic methods for improving cognitive function could not achieve the expected efficacy in same patients. Therefore, further understanding the molecular mechanism behind cognitive dysfunction in OSA disease will provide new treatment methods and targets. This review briefly summarized the clinical manifestations of cognitive impairment in OSA disease. Moreover, the pathophysiological molecular mechanism of OSA was outlined. Our study concluded that both SF and IH could induce cognitive impairment by multiple signaling pathways, such as oxidative stress activation, inflammation, and apoptosis. However, there is a lack of effective drug therapy for cognitive impairment in OSA. Finally, the therapeutic potential of some novel compounds and herbal medicine was evaluated on attenuating cognitive impairment based on certain preclinical studies.

## 1. Introduction

Obstructive sleep apnea (OSA) is a common sleep disorder characterized by intermittent hypoxia (IH) and sleep fragmentation (SF) due to upper airway collapse during sleep (Hoyos et al., 2017). Clinical studies indicate that OSA has a high incidence rate, with an estimated prevalence of 7% among adult men and 2–5% among adult women (Lumeng and Chervin, 2008). OSA patients have many symptoms related cognitive impairment, including spatial learning and memory impairment, executive function decline, and behavioral changes (Patel and Chong, 2021).

The most commonly used methods in the clinical treatment of OSA are oral appliances, surgery, and continuous positive airway pressure (CPAP) (Li et al., 2019; Toraldo et al., 2019). However, these methods have their shortcomings. First, surgical treatment must be strictly applied, as it needs to consider other factors, including apnea-hypopnea index (AHI), age, and mental state (Epstein et al., 2009). Second, although oral appliances and CPAP are commonly used practices for OSA therapy, it is ineffective in some patients. Some patients refuse to use them due to uncomfortable feelings (Carlucci et al., 2015). Finally, cognitive impairment or cognitive dysfunction could not be fully recovered in OSA patients through current therapy methods (Epstein et al., 2009). For example, one clinical trial described that most of scores neuropsychological tests for did not significantly improve after CPAP treatment in OSA patients (Bardwell et al., 2001). It is consistent with other RCTs demonstrating that CPAP treatment did not depict overall beneficial cognitive effects (Hui et al., 2000; Lim et al., 2007). Therefore, an in-depth understanding of the molecular mechanism of cognitive dysfunction in OSA disease will provide new treatment methods and targets. This article reviews the progress mechanism of cognitive dysfunction in OSA patients and summarizes some novel compounds and herbal medicine for treating cognitive impairment due to OSA.

## 2. Disease definition

Overnight polysomnography (PSG) is the standard diagnostic test for obstructive sleep apnea (St Louis, 2010). During PSG, electroencephalogram, electrooculogram, electromyogram, oronasal airflow, and oxyhemoglobin saturation can identify sleep stages, airflow, respiratory effort, body position, limb movements, ECG, and oxygen saturation. Whether the pharyngeal collapse is completely blocked or not, it is called sleep-related apnea and hypopnea, respectively (Jordan et al., 2014). An apnea is the complete cessation of airflow for at least 10 s. Hypopnea is defined as airflow reduction. “Obstructive” means breathing is frequently interrupted by upper airway obstruction, and more than 90% of airflow is reduced. AHI measures the number of apneas and hypopneas per hour of sleep to assess the severity of OSA disease. Based on the guidelines of the American Academy of Sleep Medicine (AASM), AHI < 5 indicates no disease, 5 ≤ AHI < 15 depicts a mild disease, 15 ≤ AHI < 30 represents a moderate disease, and AHI ≥ 30 characterizes a severe disease form (Muraja-Murro et al., 2014).

## 3. Symptoms of cognitive impairment in OSA

Obstructive sleep apnea is commonly associated with cognitive impairments, such as attention, verbal and visual episodic memory, and executive function (Sateia, 2003; Bucks et al., 2013). A meta-analysis revealed that vigilance, motor coordination, and executive functions were significantly impaired in OSA adults, whereas intelligence, verbal, and visual perceptual abilities were unaffected (Beebe et al., 2003). Some studies have characterized attention (Aloia et al., 2004; Bubu et al., 2020; Vanek et al., 2020), episodic memory, working memory, and executive functions (Olaithe and Bucks, 2013) as the most affected cognitive domains in OSA.

### 3.1. Attention

Attention refers to the psychological abilities of people to focus on relevant stimuli. Attention processing involves multiple aspects, such as reaction time, selective attention, and divided attention (Gagnon et al., 2014). These processes are associated with midline frontal areas and dorsolateral prefrontal cortices (Muller-Oehring and Schulte, 2014). Several studies have indicated that OSA subjects have attention impairment in all aspects (Aloia et al., 2004; Bubu et al., 2020; Vanek et al., 2020). For instance, OSA patients have more lapses and longer reaction times in tasks demanding sustained attention (Mazza et al., 2005; Gelir et al., 2014; Karimi et al., 2015) while significantly less reaction time after CPAP treatment (Djonlagic et al., 2015). Furthermore, according to the Test of Attentional Performance (TAP), OSA patients manifest deficits in divided and selective attention processes (Angelelli et al., 2020; Alkan et al., 2021).

### 3.2. Executive function

Executive function is an individually controlled and conscious effort to escort the operation of various cognitive processes. These include different cognitive abilities, such as concept formation, decision-making, mental flexibility, and problem-solving. A meta-analysis reported that executive functions across multiple tasks were impaired among OSA patients (Olaithe and Bucks, 2013).

Concept formation is a high cognitive function often operationalized as transferring the matching rule to new stimuli in a matching-to-sample task (Sukova et al., 2013). Concept formation is clinically assessed using the Wechsler Adult Intelligence Scale-Revised (WAIS-R) with these subtests: information, digit span, similarities, picture completion, block design, and digit symbol. WAIS-R demonstrated that OSA subjects had poorer scores than controls on block design, digit symbol, and picture completion (Saunamaki et al., 2009a,b, 2010). However, CPAP treatment did not significantly improve neuropsychological assessment (Saunamaki et al., 2009b,^2010^).

Decision-making is reaching decisions assessed with the Iowa Gambling Task (IGT), in which participants select cards from one of four decks. IGT characterized that scores were significantly lower in patients than in controls (Daurat et al., 2013). Furthermore, a higher rate of road traffic accidents was observed in OSA patients, impairing decision-making (Udholm et al., 2022). Moreover, OSA patients are inattentive, showing reduced reaction times on choice reaction tests (George, 2004) and decreased brain activation during an attention task involving decision-making leading to mistakes while driving (Ayalon et al., 2009).

Mental flexibility is an essential executive function underlying the ability to adapt to changing situations and respond to new information. Several investigations demonstrated a significant reduction of mental flexibility in OSA subjects (Verstraeten and Cluydts, 2004; Olaithe and Bucks, 2013). Meanwhile, the speed of mental flexibility was also enhanced after CPAP treatment (Dalmases et al., 2015).

Problem-solving is evaluating and selecting a sequence of actions to achieve a goal clinically assessed by tower test with more steps for OSA patients to solve problems (Naegele et al., 1995). Additionally, a deficit of executive functions in other aspects was observed in OSA patients, including easy impulsivity, reduced processing speed, and elevated perseverance.

### 3.3. Working memory

Working memory is the cognitive system temporarily maintaining and storing information, a short-term memory. Working memory impairment is always observed in OSA (Cosentino et al., 2008; Lau et al., 2015). Although the underlying mechanism is not fully classified, it could be related to the damage of frontoparietal connectivity since complete working memory tasks recruited a frontoparietal network of brain areas (Owen et al., 2005). A neuroimaging study revealed that the functional connectivity of the frontoparietal network showed abnormality in OSA patients (Liu et al., 2022).

### 3.4. Episodic memory

Episodic memory is remembering verbal or visual information in a space-time long-term memory. Multiple tasks could assess the ability of episodic memory, such as immediate recall, total recall for multiple steps or learning, delayed recall, free recall, and auditory task. OSA patients suffered impairment in free recall, delayed free recall, and transformed auditory span (Naegele et al., 2006). Moreover, based on the visuospatial episodic memory tasks results, there was a deficit in immediate and delayed recalls (Wallace and Bucks, 2013). Although CPAP improved the immediate and delayed memory performances, it could not ameliorate all the episodic memory components (Alchanatis et al., 2004; Bucks et al., 2013).

As discussed above, multiple cognitive ability was affected in OSA patients. However, OSA prevalence varied between 11 and 71% with cognitive impairment which was affected by OSA diagnostic methods. For example, the prevalence rates of cognitive impairment in OSA were 11, 27, 59, and 71%, respectively detected by self-report, home sleep apnea testing, Berlin questionnaire and polysomnography (Mubashir et al., 2019). Furthermore, the prevalence of cognitive impairment in OSA is related with other factors, such as severity of OSA, age and gender. Patients POSSESSING moderate to severe OSA had more severe sleep disturbances and a lower score on delayed recall test than the mild OSA group (Cai et al., 2023). Moreover, age is a significant risk factor for cognitive decline. Therefore, middle-aged OSA individuals are more likely to suffer cognitive impairment than younger ones with the similar severity of OSA (Alchanatis et al., 2008; Mathieu et al., 2008). Although several previous studies have assessed the gender-specific relationship between OSA and cognitive impairment, the conclusion need more consistency. One study described that female OSA patients had a higher risk of possessing poor prospective memory (Qiu et al., 2022). Meanwhile, OSA in women significantly reduced cortical and subcortical white matter than in men (Macey et al., 2012). However, another study indicated OSA men displayed had decreased power of extensive frequency range (sigma, beta and gamma) during sleep than in women, which plays a critical role in cognition formation (Munoz-Torres et al., 2020).

## 4. Pathophysiology of cognitive impairment in OSA

The causal mechanism of cognitive impairment remains debatable, and the existing literature has been primarily descriptive rather than based on well-defined theories. SF and intermittent blood gas abnormalities have been the most immediate physiological disturbances. They are associated with the exaggerated enhancement in upper airway resistance with sleep onset in OSA patients (Lin et al., 2019). Therefore, SF and IH are the two independent factors affecting cognitive function in OSA patients (Sforza and Roche, 2012).

### 4.1. Sleep fragmentation

Sleep fragmentation refers to sleep architecture disruption in OSA disease with poor sleep efficiency in OSA patients. This included a smaller proportion of sleep period time and reduced slow-wave sleep (SWS) (Walter et al., 2011). SF in OSA patients results in significant cognitive impairments, such as decreased mental flexibility, sustained attention, and spatial memory (Stepanski, 2002; Djonlagic et al., 2014). However, the underlying mechanisms remain poorly understood. The primary theory is that SF elicits oxidative stress and cellular damage (Shamsuzzaman et al., 2003). Since increased antioxidant activity promotes brain protection against free radicals during sleep, and wakefulness, reactive oxygen species (ROS) and other oxidative stress markers could be accumulated in the brain tissue (Mamelak, 2022). A study reported spatial learning deficits in mice exposed to SF by significantly activating oxidative stress. This could be associated with NADPH oxidase activity since mice without NADPH oxidase had normal learning after SF exposure (Nair et al., 2011b). NADPH oxidase is a vital source of generating intracellular ROS. SF could induce oxidative stress by activating NADPH oxidase to impair cognition and learning ability. The activity of nitric oxide synthase (iNOS), which regulate electron flow to enhance ROS production, was also increased in the SF model (Pandey and Kar, 2018). Then, oxidative stress results in cognitive impairment by inhibiting some neurotrophic factors expression and antioxidant genes, including BDNF and Nrf-2 (Zhang et al., 2013; Lee et al., 2022). Some synapse proteins, such as growth-associated protein 43 (GAP-43), post-synaptic density-95 (PSD-95), synapsin 1 (SYN-1), and synaptophysin (SYP), were also inhibited by SF-induced oxidative stress (Farajdokht et al., 2021).

Sleep fragmentation could also trigger an inflammatory response (Mishra et al., 2022). SF induced the expression of pro-inflammatory cytokines, such as IL-1 and TNF-α (Bertrand et al., 2020). On the other hand, long-term SF could cause vascular endothelial dysfunction by enhancing the recruitment of inflammatory cells and IL-6 expression (Carreras et al., 2014). In the SF model, multiple signaling pathways were responsible for pro-inflammatory cytokines expression, such as Toll-like receptor 4 (TLR4)/myeloid differentiation primary response protein 88 (MyD88) pathway (Xu et al., 2021b), TNF-α/NF-κB pathway (Zhang et al., 2022) and p38 MAPK pathway (Cui et al., 2019). One report indicated that SF could activate some microglial expression, vital in the inflammatory response (Kaneshwaran et al., 2019). Additionally, SF induced a selective increase in pro-inflammatory M1 macrophages by enhancing the NADPH oxidase 2 (NOX2) activity (Zhang et al., 2014).

**FIGURE 1:**
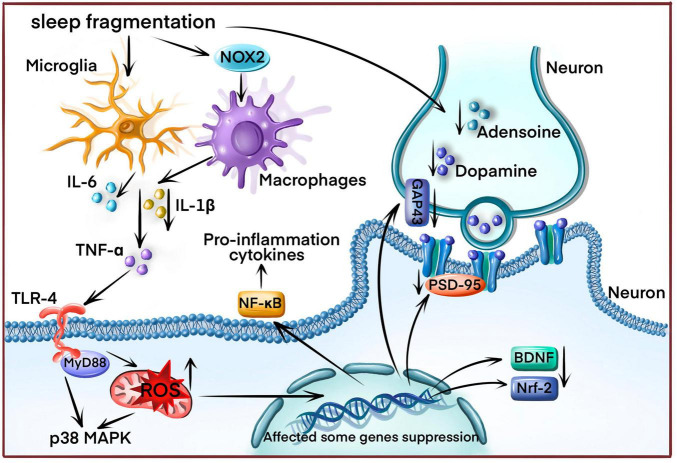
The underlying sleep fragmentation (SF) mechanism induces cognitive impairment in OSA disease. SF increases reactive oxygen species (ROS) production by enhancing NADPH oxidase activity, triggering the inflammatory response and releasing inflammatory cytokines (IL-1β, IL-6, TNF-α) from microglial and macrophages. ROS overproduction also affects various gene expressions, including neurotrophic factors, antioxidant genes, and synapse proteins. Finally, SF disrupts neurotransmitter release.

Furthermore, SF significantly reduced rapid eye movement (REM) sleep in the SF rodent model, which is associated with impairing spatial learning and the losing the NMDA receptors (Tartar et al., 2006). Therefore, the loss of the NMDA receptor could be another underlying mechanism of inducing cognitive impairment in the SF model. SF could also disrupt neurotransmitter release, such as adenosine, monoamine, and dopamine (Ramesh et al., 1999; Proenca et al., 2014). Adenosine receptor antagonists could attenuate the decline in memory-induced sleep deprivation by increasing BDNF expression in the hippocampus region (Chauhan et al., 2016). Activating the dopaminergic D2 receptor helped counteract memory impairment in the sleep deprivation model (Proenca et al., 2014; Figure 1).

### 4.2. Intermittent hypoxia

**FIGURE 2:**
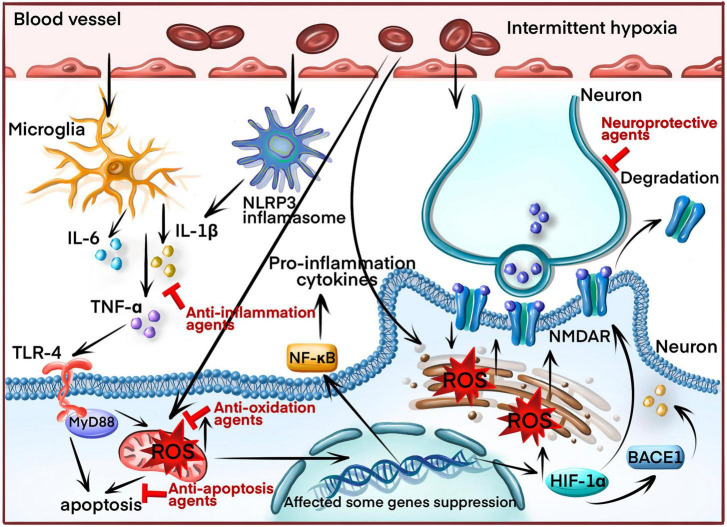
The molecular mechanism of inducing cognitive impairment in OSA patients through intermittent hypoxia (IH). IH increases reactive oxygen species (ROS) production and the nucleotide-binding domain-like receptor protein 3 (NLRP3) inflammasomes, triggering the inflammatory response and releasing inflammatory cytokines (IL-1β, IL-6, TNF-α) by activating the nuclear factor kappa B (NF-κB) signaling pathway. ROS overproduction induces apoptosis by causing mitochondrial damage and endoplasmic reticulum (ER) stress. Additionally, ROS overproduction induces the production of hypoxia-inducible factor-1α (HIF 1α) and beta-secretase 1 (BACE1). HIF-1α downregulates the N-methyl d-aspartate receptor (NMDAR) and BACE1, generating amyloid β (Aβ). Finally, the inflammatory cytokines aggravate neuronal axons, leading to synaptic damage. Blocking arrow indicated the molecular target by current therapeutic drugs.

Intermittent hypoxia (IH) is also a substantial variable associated with cognitive deficits (Dewan et al., 2015). However, IH and SF simultaneously occur in OSA, dissecting the influences of these two factors on cognitive functions, which is challenging in human subjects. An IH animal model was developed to assess the neurobehavioral effects of IH in the absence of SF, which many researchers widely accepted (Gozal et al., 2001; Row et al., 2002). Multiple mechanisms, including oxidative stress, inflammation, apoptosis, and reduction of neurotrophic factor, have been proposed to induce neurocognitive deficits due to IH (Figure 2).

#### 4.2.1. Oxidative stress

Previous studies have demonstrated increased oxidative stress in OSA, including MDA and protein carbonyl upregulation (Xu et al., 2015), excessive lipid peroxidation production (Maniaci et al., 2021) and decline of reduced glutathione (Almendros et al., 2011). Therefore, inhibiting oxidative stress is a potential therapeutic target. Under IH conditions, ROS production, as indicated by oxidative stress, is elevated due to the reduced activity of oxidoreductases in mitochondrial respiration (Xu et al., 2020). Then, elevated ROS could affect some important gene expression, such as heme oxygenase-1 (HO-1), hypoxia-inducible factor-1α (HIF-1α), and NF-κB, which also aggravate oxidative stress (Lavie, 2012). For example, stabilizing HIF-1α by IH promotes ROS synthesis in mitochondria to induce apoptosis. The inhibition of HIF-1α can reduce neuronal apoptosis (da Rosa et al., 2015). In addition, Beta-secretase 1 (BACE1), as a primary agonist to generate amyloid β (Aβ), is activated by HIF-1α. Therefore, OSA patients were highly associated with Alzheimer’s (Andrade et al., 2018). Finally, increased HIF-1α production disrupts long-term potentiation (LTP) of the hippocampus and impaired spatial memory function by downregulating the N-methyl d-aspartate receptor (NMDAR) (Arias-Cavieres et al., 2020). Thus, HIF-1α could be a potential target for future OSA therapy.

Other than altering the expression of some genes, oxidative stress could cause mitochondrial dysfunction since ROS is primarily generated in mitochondria. In IH conditions, enhanced ROS production inhibits the electron transport chain activity in mitochondria and damages mitochondrial function (Prabhakar, 2011). However, suppressing ROS production rescued the mitochondrial morphology and function in the brain (Xu et al., 2015). A previous study observed a significant correlation between OSA severity and a significant decrease in mitochondrial DNA (mtDNA) copy number in OSA patients associated with oxidative stress (Kim et al., 2014). This finding is consistent with another study that revealed that mitochondrial bioenergetics are impaired in the frontal brain regions in OSA patients (Vakulin et al., 2022).

Furthermore, ER is another region partially producing ROS. Approximately 25% of ROS are derived from the ER and are required for oxidative protein folding (Gorlach et al., 2015). Furthermore, an oxidative environment favors protein folding, particularly the formation of disulfide bonds between two cysteine residues in proteins through thiol oxidation. Therefore, increased ROS production may lead to ER homeostasis loss and accumulation of misfolded proteins. This process is called ER stress (Mello et al., 2016). Moreover, additional synthesis of misfolded or unfolded proteins could deplete glutathione (GSH) due to ER stress (Tu and Weissman, 2002). After GSH is utilized, the oxidizing environment facilitates the reoxidation of protein thiols by interacting with protein disulfide isomerase (PDI) and endoplasmic reticulum oxidoreduction (ERO-1) (Bhandary et al., 2012). These steps lead to repetitive cycles of disulfide bond breakage and formation, with each process generating additional ROS as a byproduct (Higa and Chevet, 2012). This evidence strongly implies that ER stress and ROS could reciprocally activate each other under chronic IH conditions. Increased oxidative and ER stress levels were confirmed by our previous works, contributing to the impairment of learning and memory by inducing neuronal apoptosis (Xu et al., 2015, 2021a). Thus, decreasing oxidative stress could attenuate cognitive deficits induced by hypoxia.

#### 4.2.2. Inflammation

A large number of inflammatory cytokines, such as interleukin (IL)-1, IL-6, IL-8, tumor necrosis factor-α (TNF-α), nuclear factor kappa B (NF-κB), etc., are activated in OSA patients (Liu et al., 2020). Although many factors could be implicated in the activation and progression of inflammation in OSA patients, a close relationship exists between inflammation and chronic IH (Dewan et al., 2015).

Hypoxia-inducible factor-1α, induced under IH condition, could increase NO synthesis by activating iNOS gene expression. NO is critical in initiating and regulating the inflammatory process (Abe et al., 2017). Then, excessive NO generation induced by IH could enhance neuronal apoptosis in the hippocampal CA1 region by generating lipid peroxidation (Yuan et al., 2015b). Moreover, the pro-inflammatory transcription factor NF-κB was enhanced in neutrophils and monocytes of OSA patients (Htoo et al., 2006). Meanwhile, IH treatment increased NF-kB expression in hippocampal neurons of rodent OSA model (Fei et al., 2021; Zhang C. Q. et al., 2021). However, the underlying mechanism was not elusive, with two significant explanations. One is that ROS could directly elevate NF-κB expression by activating the phosphorylation of IκBα and releasing p50 and RelA, binding to the DNA-binding domains of NF-κB and activating NF-κB transcription (Hayden and Ghosh, 2008). Another reason is that the dysregulation of leptin levels in OSA patients could increase the production of TNF-α, stimulating NF-κB activity (Berger and Polotsky, 2018).

How could these inflammatory cytokines aggravate cognitive deficits in chronic IH? There were two major theories to classify the inflammation mechanism leading to cognitive impairment under the IH condition. One is activating microglia-mediated neuroinflammation. Microglia, as inflammatory cells in the CNS, were also activated, leading to in neurocognitive and behavioral deficits caused by the IH of the animal model. IH exposure could significantly increase the density and morphological features of microglia, secreting cytokines such as IL-1β, IL-6, TNF-α, adhesion molecules, and other signaling mediators (Kiernan et al., 2016). These high cytokine levels produced by microglia can aggravate neuronal axon and synaptic damage, impairing the integrity of white matter across multiple brain regions (Hong et al., 2016). BDNF levels, crucial in neural plasticity, were decreased under IH conditions (Xie et al., 2010). Although pro-BDNF was partially released by microglia, pro-BDNF cannot change into BDNF during the persistent inflammatory phase to impair spatial memory performance (Mohammadi et al., 2020). Increasing BDNF expression can improve synaptic plasticity and decrease apoptosis caused by IH (Yin et al., 2015).

The mRNA levels of toll-like receptors-4 (TLR-4) were significantly upregulated by IH (Smith et al., 2013). Meanwhile, the monocytes from OSA patients significantly increased TLR-4 surface expression (Akinnusi et al., 2013). Therefore, TLR4 is an essential factor in IH-induced inflammation, produced by glial cells, and promotes inflammatory disorders. Glial cells are another significant category of cells activated by the IH condition (Liu et al., 2020). Then, the NF-κB signaling pathway can be activated by TLR4, enhancing the release of TNF-α and IL-1β. Moreover, TLR4 could bind with myeloid differentiation protein (MyD88) to induce cell apoptosis (Xue et al., 2017). Meanwhile, suppressing TLR4 expression could attenuate IH-induced neuronal apoptosis (Deng et al., 2015).

The nucleotide-binding domain-like receptor protein 3 (NLRP3) is a necessary inflammation interacting with procaspase-1 and apoptosis-associated speck-like protein (ASC) within the NLRP3 inflammation complex. Then, it leads to the release of caspase-1 and IL-1β (He et al., 2016). A recent study indicated that the NLRP3 inflammasome expression was increased in the brain tissue after IH treatment (She et al., 2022). Meanwhile, NLRP3 deletion elicited neuroprotection against IH treatment eliminating damaged mitochondria and reducing oxidative stress levels (Wu et al., 2021). Finally, inhibiting the NLRP3 inflammasome could suppress neuroinflammation and enhance cognitive function which was impaired by IH (Zhang et al., 2023). Therefore, NLRP3 inflammasome may be a potential target to ameliorate cognitive impairment.

#### 4.2.3. Apoptosis

Many factors involved apoptosis under IH conditions, including oxidative stress, ER stress, and inflammation response (da Rosa et al., 2015; Deng et al., 2015; Xu et al., 2015, 2021a). However, there were also other signaling pathways involved in IH-induced apoptosis. For instance, cyclic AMP response element-binding protein (CREB) activity decreased in the hippocampal CA1 after IH exposure with increased cleaved caspases-3-positive cells. Meanwhile, enhanced phosphorylation of CREB could attenuate IH-induced neurocognitive impairments by suppressing neuronal apoptosis (Wang et al., 2015). Moreover, IH-induced autophagy attenuates apoptosis by activating AMP-activated protein kinase (AMPK) and enhancing the expression levels of Bax and cleaved caspase 3. Furthermore, 3-methyladenine, as an autophagy inhibitor, could suppress these alterations (Guo et al., 2021). More factors and signaling pathways would be associated with IH-induced apoptosis with further research.

## 5. Current drug therapies for neurocognitive dysfunction in OSA patients

Presently, some drugs are adjunctive therapy for treating OSA disease, achieving good efficacy in improving cognitive impairment (Table 1).

**TABLE 1:** Summary of the drug medicine treated for OSA in clinical trials.

Name	Operation mode	Research design	Diagnosis	Treatment method		Treatment duration	Outcome	References
				Treatment group (n)	Control group (n)			
Modafinil	Oral administration	Randomized controlled trial	Polysomnography	Modafinil (200 mg/day) (77)	Placebo (80)	4 weeks	Improved performance on a test of behavioral alertness and reduced functional impairments	Dinges and Weaver, 2003
	Oral administration	Randomized controlled trial	Polysomnography	Modafinil (400 mg/day) (77)	Placebo (80)	4 weeks	Normalized daytime sleepiness, reduce the incidence of headache, nervousness	Pack et al., 2001
	Oral administration	Open-label trial	Polysomnography	Modafinil (200-400 mg/day) (58)	Placebo (67)	12 weeks	Reduced daytime sleepiness	Schwartz et al., 2003
	Oral administration	Randomized controlled trial	Polysomnography	Modafinil (100 mg/day) (9)	Placebo (11)	4 weeks	Reduced daytime sleepiness	Bittencourt et al., 2008
	Oral administration	Randomized controlled trial	Polysomnography	Modafinil (200 mg/day) (62)	Placebo (52)	4 weeks	Reduced daytime sleepiness	Inoue et al., 2013
	Oral administration	Randomized controlled trial	Polysomnography	Modafinil (400 mg/day) (30)	Placebo (30)	7 weeks	A significant improvement in alertness	Kingshott et al., 2001
Armodafinil	Oral administration	Randomized controlled trial	Polysomnography	Armodafinil (150 mg/day) (129)	Placebo (130)	12 weeks	Improved alertness, overall clinical condition, and long-term memory	Hirshkowitz et al., 2007
	Oral administration	Randomized controlled trial	Polysomnography	Armodafinil (150 mg/day) (35)	Placebo (34)	6 weeks	Improved driving safety performance and sleep quality	Kay and Feldman, 2013
	Oral administration	Randomized controlled trial	Polysomnography	Armodafinil (150 mg/day) (133), Armodafinil (250 mg/day) (131),	Placebo (130)	12 weeks	Improved sleep latency	Roth et al., 2006
	Oral administration	Randomized controlled trial	Polysomnography	Armodafinil (200 mg/day) (20)	Placebo (19)	2 weeks	Reduced sleepiness, improved the performance on standardized memory and attention	Greve et al., 2014
Atomoxetine and Oxybutynin	Oral administration	Randomized controlled trial	Polysomnography	Atomoxetine (80 mg/day) and Oxybutynin (5 mg/day) (10)	Placebo (10)	1 days	Reduced the number of obstructive events, improved the overnight oxygen desaturation and enhanced the genioglossus muscle activity	Taranto-Montemurro et al., 2017b
	Oral administration	Randomized controlled trial	Polysomnography	Atomoxetine (80 mg/day) and Oxybutynin (5 mg/day) (7)	Placebo (7)	7 days	Improved the measures of upper airway collapsibility, increased breathing stability, and slightly reduced the arousal threshold	Taranto-Montemurro et al., 2020
Fluticasone	Nasal spray	Randomized controlled trial	Polysomnography	Fluticasone (55 μg) (40)	Placebo (40)	90 days	Decreased rhinorrhea and congestion symptoms	Segsarnviriya et al., 2021
Fluticasone and montelukast	Nasal spray (Fluticasone) and Oral (montelukast)	Randomized controlled trial	Polysomnography	Fluticasone (55 μg) (13)	Placebo (13)	12 weeks	Increased sleep time and percent of REM sleep	Smith et al., 2019
Budesonide	Nasal spray	Randomized controlled trial	Polysomnography	Budesonide (64 μg) (18)	Placebo (25)	6 weeks	Improves sleep latency, slow-wave sleep, and REM sleep	Kheirandish-Gozal and Gozal, 2008
Tiagabine	Oral administration	Randomized controlled trial	Polysomnography	Tiagabine (12 mg) (7)	Placebo (7)	3 days	Increased slow-wave sleep	Taranto-Montemurro et al., 2017
γ-hydroxybutyrate	Oral administration	Self-controlled trial	Polysomnography	γ-hydroxybutyrate (12 mg) (8)	Placebo (8)	3 days	Increase in slow-wave sleep and non-REM sleep time	Series et al., 1992

### 5.1. Modafinil

Modafinil is a novel wake-promoting agent that improves wakefulness in various clinical models. The American Academy of Sleep Medicine has recommended modafinil as a ‘golden standard’ treatment for this patient population (Littner et al., 2001). Modafinil is used as an adjunct therapy in OSA disease. A randomized, double-blind, placebo-controlled trial indicated that modafinil improved performance on behavioral alertness tests and reduced functional impairments in OSA patients assessed using the psychomotor vigilance task (PVT) and the Functional Outcomes of Sleep Questionnaire (Dinges and Weaver, 2003). Meanwhile, modafinil also reduces the incidence of adverse events, including headaches, nervousness (Pack et al., 2001), and daytime sleepiness (Schwartz et al., 2003; Bittencourt et al., 2008; Inoue et al., 2013). Although modafinil did not affect sleepiness measured by the Epworth Sleepiness Scale or the Multiple Sleep Latency Test, a significant improvement in alertness was observed on the Maintenance of Wakefulness Test (Kingshott et al., 2001).

### 5.2. Armodafinil

Armodafinil is the (R)-enantiomer of the wake-promoting compound modafinil, approved for treating excessive sleepiness, OSA, and shift work disorder (Nishino and Okuro, 2008). In this 12-week, randomized, double-blind study, armodafinil significantly enhanced episodic secondary memory, patient-estimated wakefulness, and decreased fatigue with fewer adverse events (Hirshkowitz et al., 2007). Furthermore, armodafinil enhanced simulated driving safety performance in OSA patients awaiting CPA therapy (Kay and Feldman, 2013). Although the underlying mechanism is elusive, one study indicated that it could be related to increased sleep latency (Roth et al., 2006). However, no significant differences were observed in armodafinil treatment for 2 weeks compared with the placebo group according to the 2-back working memory task. Meanwhile, the neuroimaging study also indicated that armodafinil could not improve functional magnetic resonance imaging (fMRI)-measured functional brain activation (Greve et al., 2014). Another clinical trial also described that 6 months of armodafinil treatment could not improve driving task performance but affected weight loss (Chapman et al., 2018). Therefore, armodafinil did not improve all the cognitive ability aspects.

### 5.3. Anti-inflammatory drugs

Inflammation is essential in cognitive impairment, with some anti-inflammatory drugs used in clinical trials. Though AHI and rhinorrhea symptoms in OSA patients significantly decreased after treatment with intranasal fluticasone propionate, a common corticosteroid, cognitive function was not assessed (Kiely et al., 2004; Segsarnviriya et al., 2021). Montelukast, a leukotriene receptor antagonist, also affects reducing AHI (Goldbart et al., 2012). Although fluticasone and montelukast did not decrease AHI in one study, total sleep time and percent of rapid eye movement (REM) sleep were significantly elevated (Smith et al., 2019). Additionally, intranasal budesonide, another effective anti-inflammatory drug, enhances sleep latency, SWS, and REM sleep among children (Kheirandish-Gozal and Gozal, 2008). However, these clinical trials did not reveal the effect of anti-inflammatory drugs on cognitive function, which needs further investigation.

### 5.4. Atomoxetine

Atomoxetine is a selective norepinephrine reuptake inhibitor reducing hypoglossal motoneuron excitability by blocking G-coupled inwardly rectifying the potassium channels (Taranto-Montemurro et al., 2017a). Oxybutynin is an antimuscarinic with mixed effects on suppressing exceeding nicotinic excitation (Liu et al., 2005). One study demonstrated that a combination of atomoxetine with oxybutynin could decrease the number of obstructive events, enhance the overnight oxygen desaturation, elevate the genioglossus muscle activity, and reduce AHI (Taranto-Montemurro et al., 2019, 2020). However, the effect of atomoxetine and oxybutynin on reducing cognitive function is still unknown.

### 5.5. Tiagabine

Tiagabine is a γ-aminobutyric acid (GABA) reuptake receptor inhibitor increasing GABA concentration at the synaptic level of the central nervous system. Tiagabine enhanced slow-wave activity (SWA) (Taranto-Montemurro et al., 2017b) with a crucial cognitive role (Wilckens et al., 2018). On the other hand, γ-hydroxybutyrate derived from GABA also increases SWS and REM sleep time (Series et al., 1992).

### 5.6. Desipramine

Desipramine is a common tricyclic antidepressant reducing the sleep-related loss of genioglossus activity and improving pharyngeal collapsibility (Taranto-Montemurro et al., 2016a). A placebo-controlled, double-blind, randomized trial described that desipramine could mitigate the sleep-related loss of muscle activity and AHI (Taranto-Montemurro et al., 2016b). There is direct evidence to enhance the protective effect on rescuing the cognition ability of OSA patients. However, some literature depicts that desipramine improves working memory (Clinton et al., 2006; Wang et al., 2016). The impact of desipramine on improving cognition ability in OSA requires further investigation. Furthermore, other neurological drugs, such as physostigmine and mirtazapine, could reduce the AHI in diabetes patients. However, these studies did not investigate whether these drugs could improve cognitive function (Hedner et al., 2003; Carley et al., 2007).

## 6. The therapeutic mechanisms of other agents to attenuate cognitive impairment due to OSA

A few drugs were used to ameliorate cognitive deficits induced by OSA. However, experimental studies on OSA animal models indicate that chemical substances and natural products from Chinese herbs improve cognitive impairment (Table 2). Based on the action and molecular target mechanisms, these compounds are divided into: anti-oxidative properties, anti- inflammatory effects and anti-apoptosis effects.

**TABLE 2:** The mechanisms by which some chemical substances and herbs medicine treat cognitive impairment in OSA animal model.

Property	Drug name	Type of study	Treatment method		Treatment duration	Outcomes	Targets or pathways	References
			Experiment group	Control group				
Anti-oxidation	EPO	Intermittent hypoxia in mice (a cyclical pattern of 5.7% and 21% oxygen every 90 s)	EPO (5000 IU/kg/day, i.p., *n* = 24)	PBS i.p. injection (*n* = 24)	12 h/day for 14 days	Improved spatial learning and attenuated oxidative stress	Elevated levels of NADPH oxidase expression	Dayyat et al., 2012
		Intermittent hypoxia in rat (a cyclical pattern of 5.7% and 21% oxygen every 90 s)	EPO (500, 1000 IU/kg/day, i.p., *n* = 10)	PBS i.p. injection (*n* = 10)	12 h/day for 6 weeks	Improved spatial learning and attenuated oxidative stress	Increased glutathione levels and glutathione peroxidase activity	Al-Qahtani et al., 2014
	Edaravone	Intermittent hypoxia in rat (a cyclical pattern of 6% and 21% oxygen every 120 s)	Edaravone (5 mg/kg/day, i.p., *n* = 20)	Saline injection (*n* = 20)	8 h/day for 4 weeks	Attenuated IH-induced cognitive impairment	Upregulated the expression of SOD and p-CREB	Ling et al., 2020
	Hu A	Intermittent hypoxia in mice (5% and 21% oxygen 20 times/h)	Hu A (0.1 mg/kg/day, i.p., *n* = 10)	Empty liposomes (*n* = 10)	8 h/day for 3 weeks	Improved cognitive impairment and resisted oxidative stress	Activated the PKAα/Erk/CREB/BDNF signaling pathway	Yang et al., 2023
	GH	Intermittent hypoxia in rat (a cyclical pattern of 10% and 21% oxygen every 90 s)	GH (50 μg/kg/day, s.c., *n* = 8)	Vehicle injection (*n* = 8)	12 h/day for 2 weeks	Attenuated IH-induced cognitive deficits	Increased the expression of IGF-1, EPO and VEGF	Li et al., 2011
	JI-34	Intermittent hypoxia in mice (a cyclical pattern of 5.7% and 21% oxygen every 90 s)	JI-34 (50 mg/kg/day, s.c., *n* = 12)	0.1% DMSO in 10% aqueous propylene glycol solution (*n* = 12)	12 h/day for 3 weeks	Improved neurocognitive deficits, anxiety, and depression	Increased the expression of HIF-1α and EPO	Nair et al., 2013
	SFN	Intermittent hypoxia in mice (a cyclical pattern of 10% and 21% oxygen every 90 s)	SFN (0.5 mg/kg/day, i.p., *n* = 10)	Saline injection (*n* = 10)	7 h/day for 4 weeks	Decreased memory errors and apoptosis	Downregulated cleaved PARP, cleaved caspase 3, and upregulated Bcl-2 and Nrf2	Li et al., 2022
Anti- inflammation	Sesamol	Intermittent hypoxia in rat (a cyclical pattern of 10% and 21% oxygen every 120 s)	Sesamol (20 mg/kg/day, i.p., *n* = 15)	Saline injection (*n* = 15)	8 h/day for 2, 4, 6,8 weeks	Improved spatial learning assessed by Morris water maze	Reduced the levels of TNF-α and IL-1β	Zhang P. et al., 2021
Anti-apoptosis	TUDCA	Intermittent hypoxia in mice (10% and 21% oxygen every 90 s)	TUDCA (100 mg/kg/day, i.p., *n* = 15)	PBS injection (*n* = 15)	8 h/day for 2, 4 weeks	Improved spatial learning and reduced apoptosis	Inhibited endoplasmic reticulum stress	Xu et al., 2015
	Apocynin	Intermittent hypoxia in rat (a cyclical pattern of 10% and 21% oxygen every 90 s)	Apocynin (3 mg/kg/day, i.g., *n* = 10)	Saline injection (*n* = 10)	10 h/day for 4 weeks	Improved spatial learning and reduced apoptosis	Inhibiting NADPH oxidase	Hui-guo et al., 2010
	Hu A	Intermittent hypoxia in mice (9% and 21% oxygen 20 times/h)	Hu A (0.1 mg/kg/day, i.p., *n* = 10)	Empty liposomes (*n* = 10)	8 h/day for 3 weeks	Improved cognitive impairment and reduced apoptosis	Increased Bcl-2 and inhibited caspase-3 cleavage	An et al., 2020
	PCA	Intermittent hypoxia in rat (6% and 21% oxygen every 120 s)	PCA (15 mg/kg/day, i.p., *n* = 15)	Saline (*n* = 15)	8 h/day for 3 weeks	Enhanced learning and memory ability	Increased the expression of Bcl-2, BDNF and pro-BDNF	Yin et al., 2015
	SMD	Intermittent hypoxia in mice (9% and 21% oxygen every 90 s)	SMD (5.265 g/kg/day, i.g., *n* = 12)	Saline (*n* = 12)	8 h/day for 35 days	Enhanced learning and memory ability	Increased the expression of PSD-95 and BDNF	Zhao et al., 2021
	NBP	Intermittent hypoxia in rat (9% and 21% oxygen every 90 s)	NBP (80 mg/kg/day, i.g., *n* = 12)	Vegetable oil (*n* = 12)	8 h/day for 2 weeks	Enhanced learning and memory ability	Activated SIRT1/PGC-1a signaling pathway	Min et al., 2014

APOE, apolipoprotein E; DAPT, N-[N-(3,5-difluorophenacetyl)-Lalany]-S-phenylglycine t-butyl ester; DSS, dextran sodium sulfate; EPO, erythropoietin; GH, growth hormone; i.g.: intragastrically; i.p.: intraperitoneally; NBP, Dl-3n-Butylphthalide; PCA, Protocatechuic acid; p-CREB, phosphorylated-cAMP response element-binding; s.c.: subcutaneously; SFN, Sulforaphane; SMD, Shashen-Maidong Decoction; SOD, superoxide dismutase; TUDCA, Tauroursodeoxycholic acid.

### 6.1. Anti-oxidative property

Erythropoietin (EPO), a prototypic cytokine and hypoxia-sensitive gene, has been implicated in improving cognitive ability through multiple signaling pathways (Sanchez et al., 2009; Dayyat et al., 2012). For instance, mice treated with exogenously administered erythropoietin (EPO) had protection from IH-induced spatial learning deficits caused by attenuating oxidative stress responses and suppressing NADPH oxidase expression (Dayyat et al., 2012). Another study indicated that this beneficial effect elevated glutathione levels and glutathione peroxidase activity (Al-Qahtani et al., 2014).

Edaravone is another potent free radical scavenger used to treat acute attacks of cerebral infarction and improve neurological symptoms with cognitive impairment. Edaravone attenuated IH-induced cognitive impairment and elevated the number of mitochondria by upregulating the expression of SOD and phosphorylated-cAMP response element-binding (p-CREB) (Ling et al., 2020).

One study revealed Huperzine A (Hu A) elevated T-SOD and GSH-Px abilities and reduced MDA content to resist oxidative stress damage with PKAα/Erk/CREB/BDNF signaling pathway (Yang et al., 2023).

Growth hormone (GH) modulates memory and cognitive functions and is impaired in OSA (Gianotti et al., 2002). GH could attenuate IH-induced cognitive deficits by elevating the expression of IGF-1, EPO, and VEGF (Li et al., 2011). GH secretion is controlled by growth hormone-releasing hormone (GHRH) (Schussler et al., 2006). JI-34 is an agonist of GHRH, attenuating IH- induced neurocognitive deficits. The underlying mechanism is associated with increased expression of HIF-1α and EPO (Nair et al., 2013).

Sulforaphane (SFN) is extracted from cruciferous vegetables of the Brassica genus, exerting neuroprotective effects by activating autophagy or transcription factor Nrf2 (Uddin et al., 2020). SFN treatment ameliorated neurocognitive dysfunction within IH mice by downregulated cleaved PARP, cleaved caspase 3, and upregulated Bcl-2 and Nrf2 (Li et al., 2022).

### 6.2. Anti-inflammatory effects

Sesamol can alleviate cognitive impairments in chronic IH-exposed rats. This beneficial effect could reduce hippocampal TNF-α and IL-1β levels (Zhang P. et al., 2021).

### 6.3. Anti-apoptosis effects

Based on our previous research, Tauroursodeoxycholic acid (TUDCA) can decrease neuronal apoptosis and enhance hippocampal synaptic plasticity by inhibiting endoplasmic reticulum stress activation (Xu et al., 2015).

Some natural products extracted from herbal medicine are also beneficial. Apocynin is a plant drug derived from *Picrorhiza kurroa*. Apocynin attenuated IH-induced spatial learning deficits and oxidative stress by inhibiting NADPH oxidase subunit p47phox mRNA and ameliorating cell apoptosis (Hui-guo et al., 2010; Yuan et al., 2015a).

Hu A is isolated from the Chinese herb *Huperzia serrata* and could cross the blood-brain barrier (BBB). Hu A could improve cognitive impairment and neuronal damage induced by IH by increasing the Bcl-2/Bax ratio and inhibiting caspase-3 cleavage (An et al., 2020).

Protocatechuic acid (PCA) is abundant in edible fruits and vegetables and is naturally present in various herbal medicine, including *Hibiscus sabdariffa* and *Salvia miltiorrhiza*. PCA could enhance learning and memory ability and alleviate oxidative stress and apoptosis in IH-treated rats by improving the expression of Bcl-2, BDNF, and pro-BDNF and reducing cleaved caspase-3 and IL-1β (Yin et al., 2015).

Shashen-Maidong Decoction (SMD) is an herbal formula with eight Chinese medicines [*Ophiopogon japonicus* (Thunb.) Ker Gawl. (9 g); *Glehnia littoralis* (A.Gray) F.Schmidt ex Miq. (9 g); *Lablab purpureus* (L.) Sweet (4.5 g); *Morus indica* L. (4.5 g); *Polygonatum odoratum* (Mill.) Druce (6 g); *Trichosanthes kirilowii* Maxim (4.5 g); *Glycyrrhiza uralensis* Fisch. Ex DC. (3 g)]. SMD treatment in a previous study improved performance assessed using the Morris Water Maze and Y-Maze test in mice exposed to IH by enhancing ERK/CREB phosphorylation and elevating PSD-95 and BDNF expression (Zhao et al., 2021).

Finally, Dl-3n-Butylphthalide (NBP) is extracted from *Apium graveolens* L with a broad spectrum of neuroprotective properties. One study described that NBP could inhibit apoptosis and promote IH-induced autophagy by activating the SIRT1/PGC-1a signaling pathway (Min et al., 2014).

## 7. Discussion

Cognitive impairment is a common symptom of OSA, irrespective of age (Vardanian and Ravdin, 2022). Neuroimaging studies depicted cerebral cortex morphology in multiple affected regions (Kizilgoz et al., 2013), with clinical impairment across various cognition aspects. Since the cognitive impairment mechanism in OSA is complex, SF and IH are significant factors activating multiple downstream signaling pathways while causing cognitive impairment (Figures 1, 2). Some signaling pathways and proteins were targeted by SF and IH, such as excessive NADPH oxidase activity (Nair et al., 2011a,b), inducing the expression of pro-inflammatory cytokines (Bertrand et al., 2020; Liu et al., 2020) and activating the TNF-α/NF-κB pathway (Berger and Polotsky, 2018; Zhang et al., 2022). These processes do not function alone and are affected by each other. For example, HIF-1α an oxidative stress marker, could activate inflammation response by inducing NO expression (Abe et al., 2017). Initially, oxidative stress is induced by impaired antioxidant capacity. Then, some pro-inflammation cytokines are generated, accelerating oxidative stress injury and triggering neuronal apoptosis.

Although some traditional methods, including oral appliances, surgery, and CPAP, have improved certain aspects of cognitive functioning, they do not fully alleviate cognitive complaints (Vardanian and Ravdin, 2022). Therefore, drug therapy could enhance treatment outcomes and be used with other therapy methods. Some drugs have beneficial effects on improving sleep quality (Taranto-Montemurro et al., 2016b,2017b). However, more large clinical trials are required to validate these findings. Meanwhile, some chemical substances and herbal medicine could improve cognitive ability in animal studies, while could become a complementary method based on successful clinical trials.

## 8. Conclusion and perspectives

Multiple cognitive aspects are affected in OSA, which current therapy cannot improve. This review summarized the randomized controlled trials of drugs for treating OSA-related cognitive impairment. Although these drugs could improve cognition, the studies have several limitations.

First, the sample size is very small for clinical studies. There is a lack of large-scale, multicenter, randomized controlled trials on drugs treating OSA-induced cognitive impairment. Second, a wide variation occurs in the characteristics of referred patients, such as age, disease history, and disease severity. Third, the drug treatment duration is short, and the prolonged effect has yet to be investigated. Fourth, there is a lack of preclinical studies examining the possible drug targets.

Future research should be directed toward these aspects to overcome these limitations. First, some large-scale, multicenter, and controlled trials are required to determine the efficacy of these drugs. Second, standardization of the clinical drug use process should be established in treating OSA-induced cognitive impairment. Lastly, additional studies should be performed in clinical trials for some novel chemical substances and herbal medicine.

## Author contributions

DH and JC wrote the manuscript. XD and LX conceptualized the research work. LX revised the manuscript. All authors read and approved the final manuscript.
